# Sensitivity Analysis of Stochastic Calculation of SCC Regarding Aggressive Environment

**DOI:** 10.3390/ma14226838

**Published:** 2021-11-12

**Authors:** Petr Lehner, Marie Horňáková, Kristýna Hrabová

**Affiliations:** 1 Department of Structural Mechanics, Faculty of Civil Engineering, VSB—Technical University of Ostrava, L. Podéště 1875, 70800 Ostrava-Poruba, Czech Republic; marie.hornakova@vsb.cz; 2 Faculty of Civil Engineering, Brno University of Technology, Veveří 331/95, 60200 Brno, Czech Republic; kristyna.hrabova@vutbr.cz

**Keywords:** concrete, fibres, stochastic analysis, sensitivity analysis, self-compacting concrete

## Abstract

Probabilistic procedures considering the durability with respect to corrosion of reinforcement caused by aggressive substances are widely applied; however, they are based on narrow assumptions. The aspects need to be evaluated both in terms of the search for suitable application of the various experimental results and in terms of their impact on the result of the stochastic assessment itself. In this article, sensitivity analysis was used as an ideal tool to prove how input parameters affect the results of the evaluation, with consideration of different types of concrete (ordinary or self-compacting with and without fibres). These concretes may be used in aggressive environments, as an industrial floor or as a part of the load-bearing bridge structure. An example of a reinforced concrete bridge deck was selected as the solved structure. The results show that in the case of a classic evaluation, a larger amount of fibre reports a lower resistance of concrete, which contradicts the assumptions. The sensitivity analysis then shows that self-compacting concrete is more sensitive to the values of the diffusion coefficient, and with the consideration of fibres, the effect is even greater.

## 1. Introduction

The driving force behind the research on new concrete-based composite materials is the reduction of greenhouse gases and the increasing of sustainability of load-bearing and other structures [1]. The improvement of the individual components’ properties [2], replacement by more environmentally friendly types of material [3,4,5], reduction of the utilised amount of reinforcing steel [6] or the application of chemical additives [7] are just a few types of the many ways of how to effectively reduce the environmental impact of concrete structures.

One of the many possibilities is self-compacting concrete (SCC), which is often used in buildings as a cover for industrial floors thanks to its very good workability [8]. The basic properties and advantages of self-compacting concrete have already been described by many researchers, e.g., in [9,10,11]. It has been proven in [12] that fibre-reinforced concrete (FRC) can also be used for the creation of load-bearing structures thanks to the lower formation of cracks on the concrete surface. Whenever there is a need for increased strength, lower thickness and/or lower shrinkage of the concrete structure, it may be suitable to apply steel fibres, because they are able to increase the concrete tensile strength and the resistance of the concrete cover [13,14,15]. The combination of the advantages of SCC and FRC creates the so-called self-compacting steel fibre-reinforced concrete (SCC-SFR). This type of concrete has undeniable advantages when used as a top layer, especially for industrial floors, and also as a cover layer of the load-bearing parts of RC bridges exposed to aggressive substances [16,17]. The selection of the amount and type of fibre depends mainly on the purpose of the application, and this problem has been the main subject of extensive research [10,18,19]. The effect of various chemicals occurring in the industry or close to the bridges causes the degradation of the concrete and thus reduces the durability of the concrete layer. Therefore, the service life of the structure is affected as well. Long-term research related to the concrete attacked by chloride compounds has been directed towards transport structures [20], structures adjacent to the sea [21], but also industrial buildings with aggressive environments.

There are analytical and numerical approaches to calculate the durability and service life of the structure. Many of them use the finite element method (FEM) in combination with probabilistic methods. Probabilistic calculations of the durability of concrete structures influenced by the aggressive substances play an important role [22,23,24]. The probabilistic FEM solution is quite common for the assessment of static properties of civil engineering structures [25,26,27]; however, it is not very common to extend it to the durability area, and therefore many questions remain open in this field of science. These solutions can be based on the simple Monte Carlo method [28,29] or one of its improved variants, which were presented, for example, in [30,31]. In the case of the larger number of input data, a sensitivity analysis is also desirable because it shows how much influence the individual parameters have on the result [32]. A widespread problem in the case of steel fibre-reinforced concrete (SFRC) has been the inappropriate dispersion and orientation of the fibre. These problems have already been addressed by many researchers [10,33,34,35].

This paper aims to provide an analysis of the durability of the concrete in an aggressive environment and subsequent sensitivity analysis of the individual input parameters. The material parameters are based on a published study of SCC with different amounts of fibres [16]. Other parameters are based on available variances and experiments [36,37,38]. It is not necessary to take the presented example from the point of view of a specified construction, but the main goal is to point out the influence of parameters on the result of stochastic assessment through sensitivity analysis. The presented results will help with the effort of acceleration of stochastic durability calculations of RF structures with respect to chloride-induced degradation. The results allow to simplify the previously presented models, and, in the case of the low influence of an input parameter, they can be neglected or introduced in a simplified way.

## 2. Material

The compositions of the mixtures are presented in Table 1 and correspond to previously published research papers [16,39,40,41].

The concretes were prepared with the cooperation of the laboratories of the Silesian University in Gliwice and the VSB Technical University of Ostrava. Concrete mixtures from a comprehensive project dealing with the durability of concrete structures were selected for the study. The reference concrete was made of ordinary Portland cement (OPC). Self-compacting concrete (SCC) was prepared without fibres and also with fibres in amounts of 1% and 2% by weight of mass in the mixtures. The fibres are made of steel, KE20/1.7 type.

The same specific production procedures were followed. A concrete collapse test was performed on all concretes, and it was proven that all mixtures belong to the same consistency class. All investigated concretes have a cylinder strength in 28 days of about 40 to 50 MPa and, therefore, could be used in a bridge structure.

A previous study [16] showed that 2% of the fibres in concrete have a negative effect on the results, and this percentage amount of fibre is not ideal; however, for stochastic evaluation, this type of concrete was also included. For durability analysis, it was necessary to obtain other parameters, such as the diffusion coefficient, the coefficient of variation related to the variance of the values during the measurement and the ageing factor, which shows how the diffusion coefficient changes over time based on the maturation of concrete. The diffusion parameter was determined using three methods, which are described in detail in [16]. The ageing coefficient was determined using the least-squares method, which is described in detail, for example, in [42,43]. The material properties necessary for the stochastic calculation are listed in Table 2.

## 3. Stochastic and Sensitivity Analysis

The numerical model for the calculation of corrosion initiation based on the finite element method was introduced earlier in [44,45]. It allows for probabilistic analysis of the reinforced concrete structure with respect to the effect of chloride ions. The structure examined in this example is a reinforced concrete slab exposed to the ingress of chlorides, and the cross-sectional dimensions are 1.0 m in width and 0.20 m high. All inputs are summarised in Table 3.

The numerical example was performed using a probabilistic approach with a number of 100,000 simulations. The Monte Carlo probabilistic solution was applied in each step of the simulation. The durability analysis was based on a comparison of the actual chloride concentration at the reinforcement level and the chloride threshold value. When the chloride threshold is exceeded, a situation arises where corrosion of the reinforcement can be initiated. The stochastic approach evaluates this at each simulation step and uses the quantity function to evaluate an estimate of the probability of failure. Probability levels of 5%, 10% and 25% were selected.

The objective of the sensitivity analysis of the model is to quantify the relative importance of the individual input parameters. This type of analysis is important mainly to reduce the space of random variables for stochastic calculation. An overview of available methods is provided in many review articles, e.g., [32,47,48,49]. A fundamentally simple system of correlation was chosen between the results of the stochastic calculation and the input values [50]. Thus, in the first instance, a linear correlation is sought that shows a high dependence (+1) of the result on the input or a low dependence (−1). The next step is to sort the correlation coefficients for each input variable into a 100% summation to determine the degree between them.

## 4. Results and Discussion

As the monitored results, the probability quantile function, the correlation coefficients between the random variables and the resulting values of the reliability function and the significance analysis of the input variables on each other were selected.

### 4.1. Probability of Corrosion Initiation

**Figure 1:**
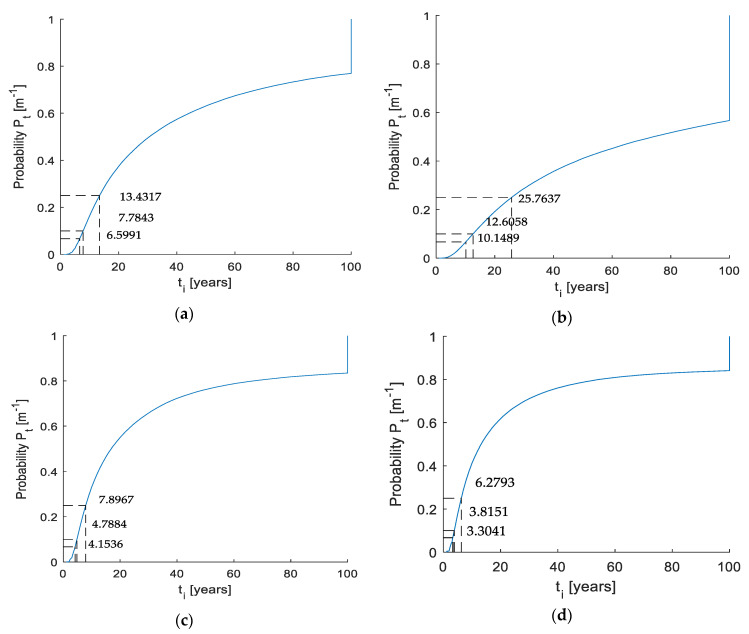
Probability of corrosion initiation is viewed as a quantity function: (**a**) OPC, (**b**) SCC 0%, (**c**) SCC 1%, (**d**) SCC 2%.

The problem studied is the probability of corrosion initiation. The quantile function of corrosion initiation for all four investigated concretes is shown in Figure 1.

Corrosion initiation represents a period when chloride penetrates the cover layer of concrete, but corrosion has not yet started in any place in the reinforcement [51,52]. As a default result, probability levels of 5%, 10% and 25% are used based on the selected design life from 0 to 100 years. It should be noted that the numerical nonstationary model was used to analyse the first 100 years of the structure’s life. If corrosion initiation is not detected, the time to exceed the limit is longer than 100 years. This is the reason why the probability values are vertical on the graphs. Table 4 summarises the results of the individual limiting probabilities and the corresponding years.

Based on the probability results, one should notice that the possibility of corrosion occurs earlier for self-compacting concrete with fibres, for both the 1% and 2% mixes. On the contrary, the probability of failure is observed to be the latest for self-compacting concrete without fibres. Standard concrete (OPC) is then worse than SCC without fibres but better than SCC with steel fibres. SCC, in general, may exhibit better diffusion properties, but conversely, fibres may increase the area bond between the concrete parts and thus negatively affect diffusion [10,53].

### 4.2. Coefficients of Correlation

**Figure 2:**
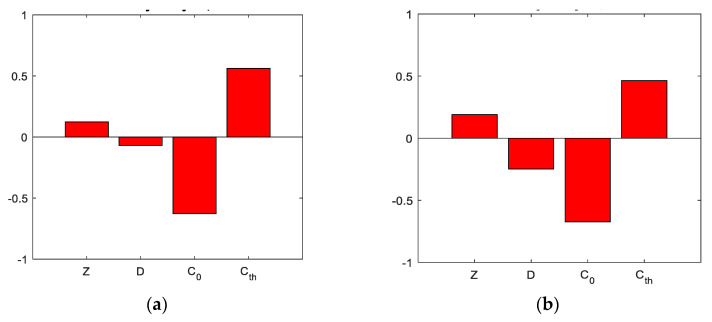
Correlation coefficients of the input parameters for all concretes: (**a**) OPC, (**b**) SCC 0%.

**Figure 3:**
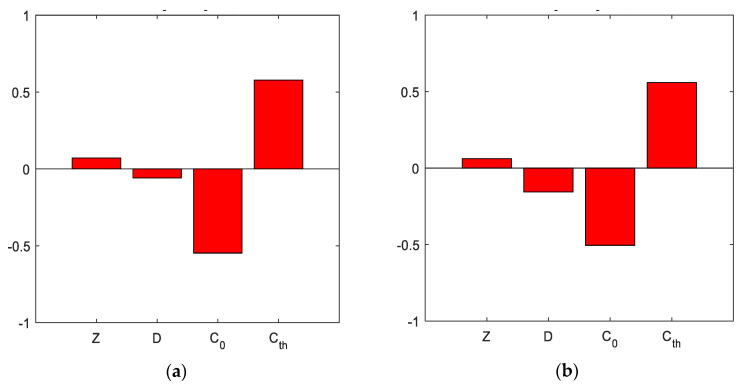
Correlation coefficients of the input parameters for all concretes: (**a**) SCC 1%, (**b**) SCC 2%.

Figure 2 and Figure 3 reveal a relationship between the chloride threshold for the initiation of corrosion (C_th_), the chloride concentration on the surface (C_0_), the depth of reinforcement (Z) and the diffusion coefficient (D) as inputs on one side, and the results of the reliability function on the other side. The chloride threshold for corrosion initiation has a significant correlation factor with the reliability function values in the case of all results. With increasing the chloride threshold, the reliability function decreases, and vice versa. For the chloride concentration at the surface, the opposite linear correlation occurs, i.e., positive and also significantly high. The concrete cover thickness and diffusion coefficient have a low correlation, and it is not possible to estimate what effect they have on the result.

### 4.3. Significance Analysis

**Figure 4:**
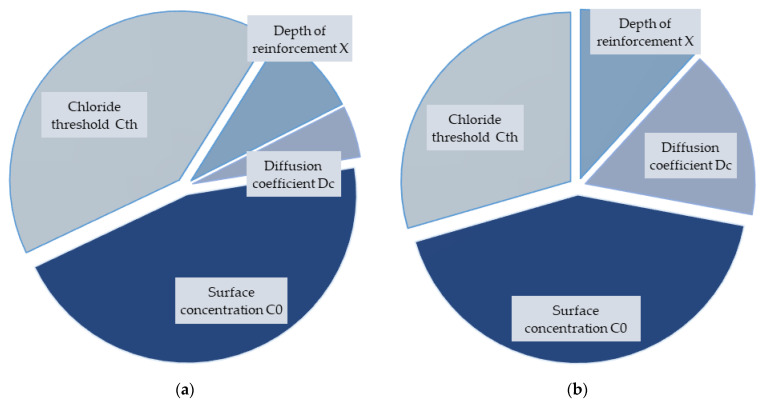
Degree of the influence of input parameters for all concretes: (**a**) OPC, (**b**) SCC 0%.

**Figure 5:**
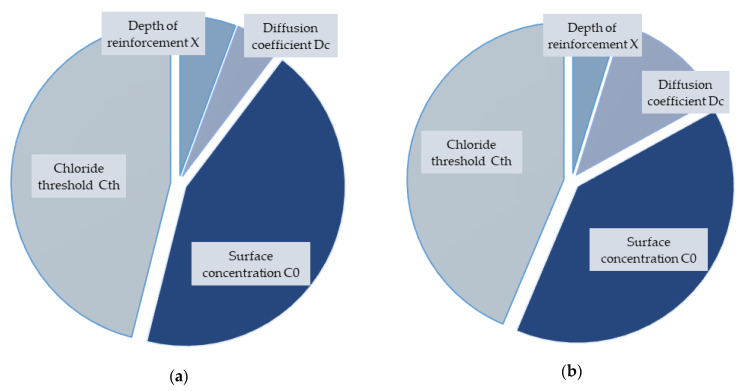
Degree of the influence of input parameters for all concretes: (**a**) SCC 1%, (**b**) SCC 2%.

The last results are from the significance analysis, which is shown in Figure 4 and Figure 5. It can be seen that the results were different for each concrete. OPC was influenced to a similar extent by the chloride threshold and surface concentration. The thickness of the concrete cover had a smaller influence, and the diffusion coefficient affected the result the least. For SCC without fibres, the results were quite different, and all variable inputs influenced the results relatively.

The opposite trend was then observed for SCC with 1% and 2% fibres, where the influence of reinforcement depth and diffusion coefficient was reduced, and even for the last mixture with 2% fibres, it is the thickness of the cover that had the least influence.

A global view of all results showed that OPC and SCC did not differ much in the evaluation of input sensitivity and significance analysis. However, the inclusion of fibres in the concrete affects the outcome differently and, therefore, needs to be taken into account in the case of other probabilistic analyses.

## 5. Conclusions

This paper provided new insights into sensitivity analysis, which is the ideal tool for demonstrating which input parameters influence or do not influence the design outcome of reinforcement failure probability with respect to aggressive environments.

The current study was limited to plain ordinary concrete and self-compacting concrete with and without fibres. This concrete can be used as a part of the load-bearing structures in aggressive environments, such as road bridges. The present findings may help to solve the problem of having to use more simulations when applying the Monte Carlo method, and therefore lower the computational power requirements. The appropriate determination of input parameters, that do not highly affect the results, may also significantly speed up the calculation. The reported results provide further evidence that different concretes and their different properties affect probabilistic analyses, and therefore, the sensitivity analysis must be an integral part of any calculation. Furthermore, the assumption that SCC reports better chloride diffusion protection properties has been confirmed and, on the contrary, fibres can negate such positive properties. Similar conclusions and hypotheses may be applied to other structural components and models. Therefore, this aspect needs to be analysed by more extensive preparation of sensitivity analysis with different concrete fibres.

## Figures and Tables

**Table 1:** Components of concrete mixtures [39].

Mixture No.	OPC	SCC 0%	SCC 1%	SCC 2%
Cement type I 42.5 R	313 kg/m^3^	490 kg/m^3^	490 kg/m^3^	490 kg/m^3^
Water	164 kg/m^3^	201 kg/m^3^	201 kg/m^3^	201 kg/m^3^
Sand	387 kg/m^3^	807 kg/m^3^	807 kg/m^3^	807 kg/m^3^
River gravel	1546 kg/m^3^	807 kg/m^3^	807 kg/m^3^	807 kg/m^3^
Super-plastificator	-	12.25 kg/m^3^	12.25 kg/m^3^	12.25 kg/m^3^
Stabilizer	-	1.96 kg/m^3^	1.96 kg/m^3^	1.96 kg/m^3^
Steel fibres	-	0 kg/m^3^	80 kg/m^3^	160 kg/m^3^
Water/cement ratio (W/C)	0.52	0.41	0.41	0.41

**Table 2:** Diffusion properties.

Mixture No.	Diffusion Coefficient (m^2^/s × 10^−11^)	Coefficient of Variation (-)	Ageing Factor (-)
OPC	1.0723	0.035	0.1344
SCC 0%	1.1575	0.107	0.2779
SCC 1%	1.7311	0.036	0.1157
SCC 2%	2.1111	0.104	0.0932

**Table 3:** Input variable parameter data for the analyses.

Parameter	Unit	Probabilistic Approach	
		Range/Value	Function
Variation coefficient, *cv_RV_*	-	−1.00–1.00	Constant
Width of the investigated cross-section, *b*	m	1.00	Constant
Height of the investigated cross-sectional surface, *h*	m	0.20	Constant
Depth of reinforcement, Z	m	0.04–0.11	Histogram [36]
Chloride threshold for corrosion initiation, *C*_th_	% weight of cement	0.09–0.51	Histogram [37]
Concentration of chloride at the surface, *C*_0_	% weight of cement	0.21–1.63	Histogram [46]
Initial concentration of chloride in the cross-section, *C*_b_	% weight of cement	0	Constant
Monitored life span, *t*	years	100	Constant
Number of simulations	-	100,000	Constant

**Table 4:** Period to the initiation of corrosion, *t*_i_ (years), for a selected probability of the initiation of corrosion.

Mixture No.	Probability of Corrosion Initiation *P_f_* (%)		
	5	10	25
OPC	6.60	7.78	13.43
SCC 0%	10.15	12.61	25.76
SCC 1%	4.15	4.79	7.90
SCC 2%	3.31	3.82	6.28

## Data Availability

Not applicable.

## References

[1] Yan L., Chouw N., Lu Y. (2014). Sustainable Concrete and Structures with Natural Fibre Reinforcements. Infrastructure Corrosion and Durability—A Sustainability Study.

[2] Sharbatdar M.K., Abbasi M., Fakharian P. (2020). Improving the Properties of Self-compacted Concrete with Using Combined Silica Fume and Metakaolin. Period. Polytech. Civ. Eng..

[3] Kocot A., Ponikiewski T. (2021). Influence of Artificial Waste Modification on Strength of Cementitious Composite. Trans. VSB—Tech. Univ. Ostrav. Civ. Eng. Ser..

[4] Stevulova N., Vaclavik V., Hospodarova V., Dvorský T. (2021). Recycled Cellulose Fiber Reinforced Plaster. Materials.

[5] Gholampour A., Ozbakkaloglu T. (2018). Time-dependent and long-term mechanical properties of concretes incorporating different grades of coarse recycled concrete aggregates. Eng. Struct..

[6] Lee D., Son S., Kim D., Kim S. (2020). Special-Length-Priority Algorithm to Minimize Reinforcing Bar-Cutting Waste for Sustainable Construction. Sustainability.

[7] Svintsov A.P., Shchesnyak E.L., Galishnikova V.V., Fediuk R.S., Stashevskaya N.A. (2020). Effect of nano-modified additives on properties of concrete mixtures during winter season. Constr. Build. Mater..

[8] Okrajnov-Bajic R., Vasovic D. (2009). Self-compacting concrete and its application in contemporary architectural practice. Spatium.

[9] Alberti M., Enfedaque A., Galvez J. (2017). Fibre reinforced concrete with a combination of polyolefin and steel-hooked fibres. Compos. Struct..

[10] Ponikiewski T., Katzer J. (2016). Fresh Mix Characteristics of Self-Compacting Concrete Reinforced by Fibre. Period. Polytech. Civ. Eng..

[11] Geiker M., Jacobsen S. (2019). Self-compacting concrete (SCC). Dev. Formul. Reinf. Concr..

[12] Kessler S., Gehlen C. Measurement Uncertainty and POD and Its Influence Remaining Service Life Evaluation. Proceedings of the 3rd International fib Congress and Exhibition, Incorporating the PCI Annual Convention and Bridge Conference: Think Globally, Build Locally, Proceedings.

[13] Abrishambaf A., Barros J.A., Cunha V.M. (2015). Tensile stress–crack width law for steel fibre reinforced self-compacting concrete obtained from indirect (splitting) tensile tests. Cem. Concr. Compos..

[14] Faraj R.H., Mohammed A.A., Mohammed A., Omer K.M., Ahmed H.U. (2021). Systematic multiscale models to predict the compressive strength of self-compacting concretes modified with nanosilica at different curing ages. Eng. Comput..

[15] Asteris P.G., Ashrafian A., Rezaie-Balf M. (2019). Prediction of the Compressive Strength of Self-Compacting Concrete Using Surrogate Models. Comput. Concr..

[16] Lehner P., Konečný P., Ponikiewski T. (2020). Comparison of Material Properties of SCC Concrete with Steel Fibres Related to Ingress of Chlorides. Crystals.

[17] Stawiski B., Kania T. (2020). Tests of Concrete Strength across the Thickness of Industrial Floor Using the Ultrasonic Method with Exponential Spot Heads. Materials.

[18] Ganesha M., Umesh S.S., Anand V.R. (2019). Research on the Strength Parameters of Poly Propylene Fiber Reinforced Concrete and Steel Fiber Reinforced Concrete. Int. J. Recent Technol. Eng..

[19] da Silva G.C.S., Christ R., Pacheco F., de Souza C.F.N., Gil A.M., Tutikian B.F. (2020). Evaluating steel fiber-reinforced self-consolidating concrete performance. Struct. Concr..

[20] Safehian M., Ramezanianpour A.A. (2015). Prediction of RC structure service life from field long term chloride diffusion. Comput. Concr..

[21] Zhuo W., Yan Q., Yang Z., Lin S., Lin K., He F. (2019). Chloride Penetration in Coastal Concrete Structures: Field Investigation and Model Development. Adv. Mater. Sci. Eng..

[22] Kožar I., Malić N.T., Simonetti D., Božić Ž. (2020). Stochastic properties of bond-slip parameters at fibre pull-out. Eng. Fail. Anal..

[23] Zambon I., Ariza M.P.S., e Matos J.C., Strauss A. (2020). Value of Information (VoI) for the Chloride Content in Reinforced Concrete Bridges. Appl. Sci..

[24] Loreto G., Di Benedetti M., De Luca A., Nanni A. (2019). Assessment of reinforced concrete structures in marine environment: A case study. Corros. Rev..

[25] Zięba J., Buda-Ożóg L., Skrzypczak I. (2019). Probabilistic method and FEM analysis in the design and analysis of cracks widths. Eng. Struct..

[26] Arregui-Mena J.D., Margetts L., Mummery P.M. (2014). Practical Application of the Stochastic Finite Element Method. Arch. Comput. Methods Eng..

[27] Vu K.A.T., Stewart M.G. (2000). Structural reliability of concrete bridges including improved chloride-induced corrosion models. Struct. Saf..

[28] Metropolis N., Ulam S. (1949). The Monte Carlo Method. J. Am. Stat. Assoc..

[29] Marek P., Brozzetti J., Gustar M., Elishakoff I. (2002). Probabilistic Assessment of Structures using Monte Carlo Simulations. Appl. Mech. Rev..

[30] Lehner P., Horňáková M., Konečný P. (2020). Numerical Approximation of Time-Dependent Chloride Diffusion Model Parameters via Probabilistic Monte Carlo Method. AIP Conf. Proc..

[31] Rao K.D., Gopika V., Rao V.S., Kushwaha H., Verma A., Srividya A. (2009). Dynamic fault tree analysis using Monte Carlo simulation in probabilistic safety assessment. Reliab. Eng. Syst. Saf..

[32] Saltelli A. (2002). Sensitivity Analysis for Importance Assessment. Risk Anal..

[33] Faifer M., Ferrara L., Ottoboni R., Toscani S. (2013). Low Frequency Electrical and Magnetic Methods for Non-Destructive Analysis of Fiber Dispersion in Fiber Reinforced Cementitious Composites: An Overview. Sensors.

[34] Zhu Y., Blumenthal W.R., Lowe T.C. (1997). Determination of Non-Symmetric 3-D Fiber-Orientation Distribution and Average Fiber Length in Short-Fiber Composites. J. Compos. Mater..

[35] Balázs G.L., Czoboly O., Lublóy É., Kapitány K., Barsi Á. (2017). Observation of steel fibres in concrete with Computed Tomography. Constr. Build. Mater..

[36] Scannell W.T., Sohanghpurwala A.A. (1998). Verification of Effectiveness of Epoxy-Coated Rebars.

[37] Darwin D., Browning J., O’Reilly M., Xing L., Ji J. (2009). Critical Chloride Corrosion Threshold of Galvanized Reinforcing Bars. ACI Mater. J..

[38] Weyers R.E., Pyc W., Sprinkel M.M. (1998). Estimating the Service Life of Epoxy-Coated Reinforcing Steel. ACI Mater. J..

[39] Lehner P., Konečnỳ P., Ponikiewski T., Katzer J., Cichocki K., Domski J. (2018). Relationship between Mechanical Properties and Conductivity of SCC Mixtures with Steel Fibres. Research and Modelling in Civil Engineering 2018.

[40] Konečný P., Lehner P., Ponikiewski T., Miera P. (2017). Comparison of Chloride Diffusion Coefficient Evaluation Based on Electrochemical Methods. Procedia Eng..

[41] Sucharda O., Lehner P., Konečný P., Ponikiewski T. (2018). Investigation of Fracture Properties by Inverse Analysis on Selected SCC Concrete Beams with Different Amount of Fibres.

[42] Lehner P., Ghosh P., Konečný P. (2018). Statistical analysis of time dependent variation of diffusion coefficient for various binary and ternary based concrete mixtures. Constr. Build. Mater..

[43] Konečný P., Lehner P., Ghosh P., Morávková Z., Tran Q. (2020). Comparison of procedures for the evaluation of time dependent concrete diffusion coefficient model. Constr. Build. Mater..

[44] Konečný P., Veselý V., Lehner P., Pieszka D., Žídek L. (2014). Investigation of Selected Physical Parameters of Cementitious Composite during Sequential Fracture Test. Adv. Mater. Res..

[45] Le T.D., Lehner P., Konečný P. (2019). Probabilistic Modeling of Chloride Penetration with Respect to Concrete Heterogeneity and Epoxy-Coating on the Reinforcement. Materials.

[46] Konečný P., Lehner P. (2016). Effect of cracking and randomness of inputs on corrosion initiation of reinforced concrete bridge decks exposed to chlorides. Frat. Ed. Integrità Strutt..

[47] Boddy A., Bentz E., Thomas M., Hooton R. (1999). An overview and sensitivity study of a multimechanistic chloride transport model. Cem. Concr. Res..

[48] Král P., Hradil P., Hušek M., Kala J., Kala Z. (2018). Sensitivity analysis and optimization as tools for the inverse concrete material model parameter identification. Proceedings of the International Conference of Numerical Analysis and Applied Mathematics (ICNAAM 2017).

[49] Kala Z., Kala J., Simos T.E., Psihoyios G., Tsitouras C., Anastassi Z. (2011). Sensitivity Analysis of Stability Problems of Steel Structures using Shell Finite Elements and Nonlinear Computation Methods. AIP Conf. Proc..

[50] Schober P., Boer C., Schwarte L.A. (2018). Correlation Coefficients. Anesth. Analg..

[51] Ghosh P., Konečný P., Tikalsky P.J. (2011). SBRA Model for Corrosion Initiation of Concrete Structures. Model. Corroding Concr. Struct..

[52] Zhang X.-G., Zhao Y.-G., Xing F., Lu Z.-H. (2011). Coupling effects of influence factors on probability of corrosion initiation time of reinforced concrete. J. Cent. South. Univ. Technol..

[53] Ponikiewski T., Katzer J., Bugdol M., Rudzki M. (2015). X-ray computed tomography harnessed to determine 3D spacing of steel fibres in self compacting concrete (SCC) slabs. Constr. Build. Mater..

